# Active Mediation of Plasmon Enhanced Localized Exciton Generation, Carrier Diffusion and Enhanced Photon Emission

**DOI:** 10.1038/s41598-017-00964-5

**Published:** 2017-04-13

**Authors:** Sharmin Haq, Sadhvikas Addamane, Bijesh Kafle, Danhong Huang, Ganesh Balakrishnan, Terefe G. Habteyes

**Affiliations:** 1 Department of Chemistry & Chemical Biology, University of New Mexico, Albuquerque, NM 87131 United States; 2 Electrical & Computer Engineering, University of New Mexico, Albuquerque, NM 87131 United States; 3 Optical Science & Engineering Program, University of New Mexico, Albuquerque, NM 87131 United States; 4 Center for High Technology Materials, University of New Mexico, Albuquerque, NM 87131 United States; 5 Space Vehicles Directorate, Air Force Research Laboratory, Kirtland AFB, NM 87117 United States

## Abstract

Understanding the enhancement of charge carrier generation and their diffusion is imperative for improving the efficiency of optoelectronic devices particularly infrared photodetectors that are less developed than their visible counterpart. Here, using gold nanorods as model plasmonic systems, InAs quantum dots (QDs) embedded in an InGaAs quantum well as an emitter, and GaAs as an active mediator of surface plasmons for enhancing carrier generation and photon emission, the distance dependence of energy transfer and carrier diffusion have been investigated both experimentally and theoretically. Analysis of the QD emission enhancement as a function of distance reveals a Förster radius of 3.85 ± 0.15 nm, a near-field decay length of 4.8 ± 0.1 nm and an effective carrier diffusion length of 64.0 ± 3.0 nm. Theoretical study of the temporal-evolution of the electron-hole occupation number of the excited states of the QDs indicates that the emission enhancement trend is determined by the carrier diffusion and capture rates.

## Introduction

Excitons and localized surface plasmons are the two fundamental excitation characteristics of nanoscale materials. The coupling between excitonic and plasmonic materials promises control of photon emission^1–3^ and creation of new metamaterial properties^4, 5^ that do not exist in nature. Fundamental understanding of exciton-plasmon interaction can lead to development of efficient photovoltaics^6–8^, photodetectors^9, 10^, photocatalysis^11, 12^ and other optoelectronic devices. Classic experiments on exciton-plasmon interactions have often used optically transparent spacer materials between the plasmonic metal and excitonic semiconductor materials^1, 3, 13, 14^. Coupling through optically transparent spacers does not allow studying charge transport process. On the other hand, studies on plasmon enhanced near-infrared photo-detectors are focused on coupling metallic two-dimensional-hole-arrays with layered semiconductor materials such as InAs/InGaAs/GaAs dot-in-a-well (DWELL) structures^9, 15^. This enhancement mechanism exploits the extraordinary optical transmission effect^16^, where the transmitted field extends to about 1 μm length covering the whole active region^15^, and does not allow fundamental understanding of localized exciton generation, charge carrier diffusion and recombination.

In this work, energy transfer and charge carrier diffusion are investigated systematically taking advantage of the tight electric field localization at the interfaces of plasmonic gold nanorods (AuNRs) and semiconductor GaAs that is grown over the InAs/InGaAs DWELL with accurate control of the GaAs thickness. When excitation energy that is above the GaAs band gap is chosen, the localized electric field enhances generation of electron-hole pairs (excitons) in a defined spatial region away from the InAs QDs so that carrier diffusion and capture rates are studied by monitoring the emission intensity of the QDs. The fact that the GaAs thickness can be controlled with sub-nanometer accuracy allows us to study the distance dependencies of near-field confinement, carrier diffusion and excitation energy transfer to the metal surface that leads to quenching of photoluminescence (PL) at short AuNR-InAs separation distances.

The choice of the QDs as opposed to InGaAs/GaAs quantum well as model system is motivated by the more efficient excitation of the QDs with in-plane polarized electric field of light at normal incidence^17–19^. In addition, QDs can facilitate the theoretical comparison as they can be treated as point dipoles. We note that the AuNR plasmon resonances that overlaps with the excitation wavelength (λ = 633 nm) are far from the emission wavelength (λ ≈ 1200 nm) of the QDs. Therefore, the PL enhancement originates purely from the enhancement of photoabsorption and exciton generation inside the GaAs and InGaAs layers. All the optical measurements have been carried out at room temperature at which the thermal energy is larger than the exciton binding energy. As a result, the excitons generated at the plasmonic hot spots of the AuNR-GaAs interface can dissociate, and the PL enhancement can be attributed to the diffusion of charge carriers (electrons and holes) to the InAs QDs. When the GaAs thickness is comparable to the near-field decay length, the near-field directly enhances the exciton generation inside the InAs/InGaAs, where electron-hole recombination may dominate over exciton dissociation because of the proximity to the emitting QDs.

## Results and Discussion

**Figure 1:**
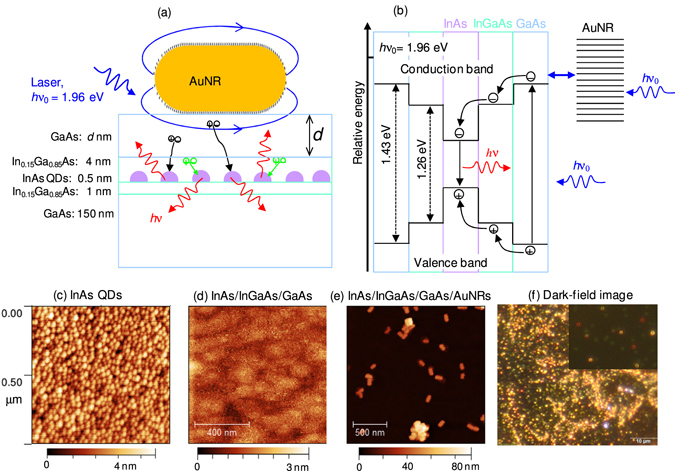
Integration of semiconductor and plasmonic materials for the studies of plasmon enhanced exciton generation and photon emission. (**a**) Schematic showing the InAs quantum dots (QDs) confined in an InGaAs quantum well, capped with GaAs of variable thickness (*d*) and coupled to a single gold nanorod (AuNR). The short lines around the colloidal AuNR represent the surface ligands (cetyltrimethylammonium bromide). The plasmon near-field enhances electron-hole pair generation in the GaAs and InGaAs layers and the enhancement of photon emission by the InAs QDs depends on the carrier capture rates from the GaAs (black arrow) and from the InGaAs well (green arrow) by the QDs. (**b**) The energy level diagram shows that the excitation energy (1.96 eV) is high enough to promote electron from the valence band to the conduction band in any of the materials including the GaAs that has the highest band gap energy. (**c**–**e**) Topographic AFM scan images obtained before the InGaAs and GaAs layers are grown (**d**), after the InGaAs and GaAs layers are grown (**e**), and after the AuNRs are drop-casted on the GaAs surface. (**f**) Dark-field image of AuNRs on GaAs surface. The color of the dark-field images of the individual AuNRs varies from red to green, depending on the proximity of the AuNRs to the GaAs surface.

The interfacial and energetic structures of the integrated plasmonic and semiconductor materials are illustrated in the schematic shown in Fig. 1 (see Method for the details of the fabrication and integration procedures). The spacing between the GaAs and the AuNR surfaces can vary depending on the amount of surface ligands (represented by short lines in Fig. 1a) on the colloidal AuNRs. The size of the AuNRs (nominal size: 40 nm diameter and 80 nm length; see ref. 20 for the size distribution) is chosen so that the plasmon resonances overlap with the 633 nm excitation wavelength. As illustrated in Fig. 1b, the excitation energy of the laser (*hc*/λ = 1.96 eV, where *h* is Planck’s constant and *c* is the speed of light) is above the interband electronic transition energies of both GaAs (~1.43 eV) and InGaAs (~1.26 eV) materials. Excitation of the localized surface plasmon resonances of the AuNRs interfaced with the GaAs further enhances the exciting electric field and improves the efficiency of exciton generation. The topographic atomic force microscope (AFM) image in Fig. 1c shows that our molecular beam epitaxy crystal growth procedure produces close-packed self-assembled InAs QDs. The growth of the InGaAs and GaAs layers results in a planar surface as seen in Fig. 1d. The topographic image of the region, where an aqueous solution of gold nanorods is drop-casted on the GaAs surface shows randomly distributed individual AuNRs and some aggregates as shown in Fig. 1e. The distribution of the AuNRs over a larger area is displayed in the diffraction limited dark-field scattering image in Fig. 1f. In agreement with previous observations on silicon substrate^21^, the dark-field images of individual AuNRs on GaAs surface have doughnut shaped structures. Interestingly, the color of the scattering images of the individual AuNRs ranges from red to green (see the inset image in Fig. 1f), which can be sorted as red, orange, yellow and green (ROYG).

**Figure 2:**
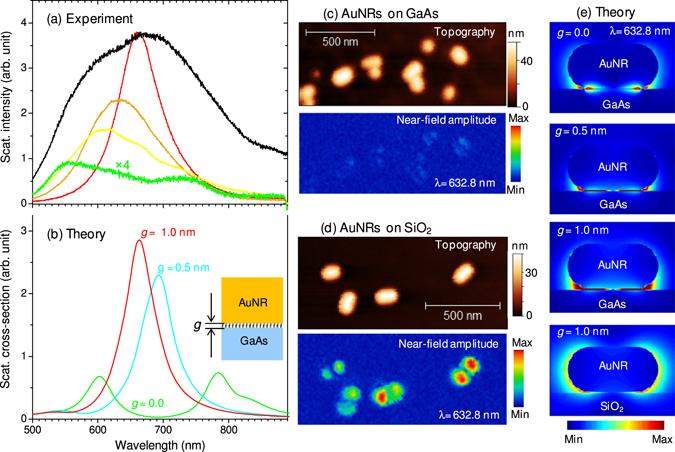
Optical properties of colloidal AuNRs deposited on GaAs surface. (**a**) Scattering spectra of individual AuNRs that appear red, orange, yellow and green in the dark-field image (shown with the corresponding colors) along with the scattering spectrum of collection of AuNRs (black curve). The black curve is recorded at lower acquisition time to avoid detector saturation and the intensity is adjusted to match its maximum to that of the red spectrum. (**b**) The calculated scattering cross-section of AuNR (40 nm × 80 nm) when the AuNR-GaAs gap is 0 (green line), 0.5 nm (cyan line) and 1.0 nm (red line). (**c**) Topography and measured near-field amplitude of AuNRs on GaAs surface. (**d**) Similar to (**c**) but the AuNRs are supported on silica surface. The results in (**c**) and (**d**) are obtained with the same experimental conditions and the near-field amplitudes are scaled to the same maximum. (**e**) The near-field amplitude calculated on a plane that cuts the AuNR through its center vertically for AuNR-GaAs separation distances of 0.0, 0.5 and 1.0 nm as labeled, compared to the field distribution when the AuNR is supported on a silica surface (bottom most panel).

Single particle scattering spectra have been recorded from the particles that appear red, orange, yellow and green in the dark-field image, and representative results are presented in Fig. 2a (using the corresponding line color), along with the scattering spectrum of ensemble of the AuNRs (black curve). The plasmon scattering spectra of the red particles have 666 ± 17 nm peak wavelength and 81 ± 5 nm full-width-at-half maximum (FWHM), compared to 628 nm average peak wavelength and 52 nm FWHM for the same size AuNRs on a glass substrate, which have been characterized under the same optical settings. The significant red-shift and broader FWHM of the AuNR resonances on GaAs compared to that on glass, can respectively be attributed to the high refractive index and the dissipative property of the GaAs substrate as can be realized from its dielectric function (ɛ_GaAs_ = 14.83 + i1.52 at 632.8 nm excitation wavelength). As seen in Fig. 2a, the spectra are progressively broadened as the color changes from red to orange and to yellow. Finally spectral splitting is observed for the green particles that have very weak overall scattering intensity. The theoretical scattering spectra (Fig. 2b), calculated using finite-difference time domain (FDTD) method of electromagnetic simulation, agree with the measured spectra (Fig. 2a) of the red particles when there is 0.5 to 1.0 nm gap between the AuNR and GaAs surfaces. The spectra of the green particles is reasonably reproduced in the simulation when the AuNR is in direct contact with the GaAs surface as shown by the green curve in Fig. 2b: the longitudinal plasmon resonance shifts to the red by about 150 nm with respect to that supported on glass surface, and a substrate-induced plasmon resonance^21, 22^ appears around 600 nm. Based on this comparison of experimental and theoretical results, the ROYG color can be attributed to different proximity of the AuNRs to the GaAs, R being the furthest from the surface and G the closest. The variation in the separation distances can mainly be attributed to different amounts of the surface ligands (cetyltrimethylammonium bromide) on the colloidal gold nanorods. In our study of plasmon enhanced exciton generation and diffusion, the illumination area is relatively large (~3 mm^2^), and therefore, the enhancement results from ensemble averaging represented by the black spectrum in Fig. 2a.

High resolution electric field imaging using our apertureless near-field scanning optical microscope (ANSOM)^23, 24^ shows very weak near-field amplitude on the gold nanorods when supported on GaAs (Fig. 2c), compared to the large near-field amplitude of the dipolar mode for the AuNRs supported on a silica surface (Fig. 2d), which shows orientation dependent near-field optical response in agreement with our previous results^20^. This weaker near-field amplitude for AuNR-GaAs than for AuNR-SiO_2_ is in contrast to our observation of a much stronger scattering intensity for AuNRs on GaAs than for those on silica surfaces. The results of the FDTD simulation displayed in Fig. 2e shows that the electric field is tightly localized at the AuNR – GaAs interfacial region. This tight electric field localization is significantly different from that observed when the AuNR are supported on the SiO_2_ substrate, where the electric field amplitude at the AuNR-SiO_2_ interface is comparable to the amplitude on the top surface (AuNR-air interface). This drastically different field localization results in weaker near-field amplitude in the ANSOM data for the AuNRs on the GaAs because the electric field localized at the AuNR-GaAs interfacial region is about 40 nm (the nominal diameter of the AuNRs) away from the near-field probing tip, and it is inaccessible by the ANSOM imaging technique. However, this electric field localization is advantageous for enhancing exciton generation in the GaAs layer very close to the interface and for studying energy transfer and carrier diffusion as discussed next.

**Figure 3:**
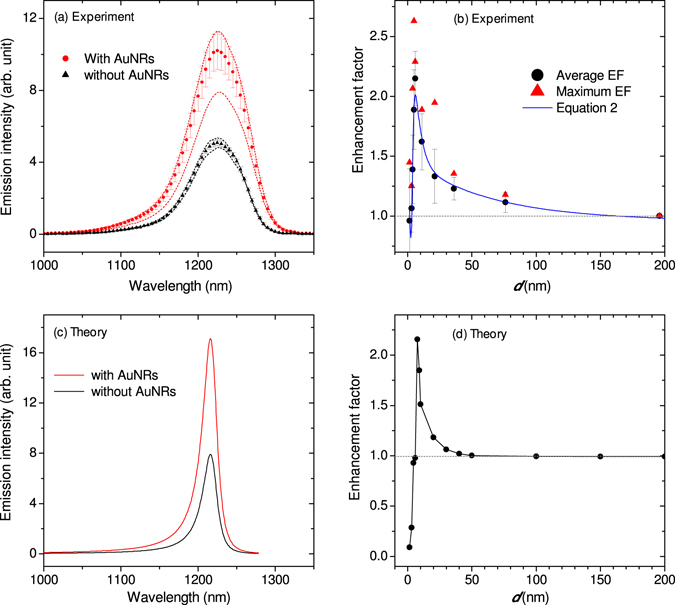
Emission enhancement and distance dependence. (**a**) Photoluminescence acquired from different locations where there are no AuNRs (black lines) and where there are AuNRs (red lines) for 6 nm GaAs thickness (*d*) as an example. (**b**) Integrated intensity ratio (enhancement factor) as a function of *d*. The error bars on the average data points (black circles) represent one standard deviation. The maximum enhancement factors (red triangles) represent the ratios of the integrated intensities of the highest intensity spectra obtained in the presence and absence of AuNRs for different GaAs thicknesses. The solid blue line is obtained by fitting equation (2) to the average data. (**c**) Calculated emission spectra of InAs QDs for *d* = 7.5 nm in the presence (red line) and absence (black line) of AuNRs. (**d**) Calculated enhancement factor (ratio of emission intensities) plotted as a function of *d*.

The PL enhancement/quenching as a function of GaAs thickness *d* (as defined in Fig. 1a) is studied by comparing the PL intensity (*I*_*InAs*/*AuNR*_) of the region where the AuNRs are deposited to the intensity (*I*_*InAs*_) of the region where there are no AuNRs. Representative PL spectra of the two regions are compared in Fig. 3a considering a 6 nm GaAs thickness. In each case, 15 to 20 spectra are acquired by irradiating different areas in the respective regions, and the spectra with minimum and maximum peak intensities along with the average data are plotted in Fig. 3a. In the absence of the AuNRs, no significant intensity fluctuation is observed as indicated by a standard deviation that is not much larger than the symbols (black triangle, in Fig. 3a), indicating the uniformity of the InAs QD number density within the irradiation volume. Comparing the red and black curves in Fig. 3a, it can be seen that the weakest PL intensity obtained in the presence of the AuNRs is significantly stronger than the highest PL intensity obtained from the region without the AuNRs. The intensity fluctuation for the regions where there are AuNRs (red lines) is due to the difference in the number density and aggregation of AuNRs at different locations. However, this intensity fluctuation is minor considering the spatial variation of the gold nanorod orientation and number density at the nanoscale as shown in Fig. 1e. The reproducibility of the relative intensity at different locations is due to a large illumination area that provides statistical representation of the distribution of orientation and aggregation.

Similar comparison as presented in Fig. 3a has been repeated for *d* = 1.5 nm to 196 nm and the results are discussed in terms of an emission enhancement factor (*EF*) as follows. The *EF* for the samples with different GaAs thicknesses is evaluated independently by calculating the integrated PL intensity ratio as follows.

1 $$EF=\frac{{I}_{InAs/AuNR}}{{I}_{InAs}}$$

Both the average (black circles) and maximum (red triangles) *EF* values are plotted in Fig. 3b. As seen in the figure, the enhancement factor first increases exponentially with decreasing distance until *d* = 6 nm and then decreases rapidly for shorter distances. The general trend is in good agreement with the observation of fluorescence enhancement and quenching by a gold nanosphere reported by Novotny and co-workers^14^. However, unlike in the previous report (where the spacing medium is air), in our study the emitter and the plasmonic nanostructures are separated by GaAs, whose dielectric and absorption property at the excitation wavelength can change the enhancement mechanism fundamentally.

The results presented in Fig. 2e indicates a very short decay length of the plasmon near-field into the GaAs, which results in enhanced electron-hole generation close to the AuNR-GaAs interface. Based on the observation in Fig. 3b and the tight near-field localization at AuNR-GaAs interface, the net emission enhancement is expected to involve the following distance dependent processes: (i) Förster energy transfer – at short distances, excitation energy transfer from the QDs to the metal surfaces of AuNRs can be the dominant process, which results in reduced emission intensity, (ii) near-field enhanced electron-hole generation inside the InGaAs and efficient carrier capture by the QDs, and (iii) enhanced electron-hole generation at the AuNR-GaAs interface, carrier diffusion through the bulk GaAs and capture by InAs/InGaAs. To extract the length scales of these processes from the experimental enhancement factor (*EF*) presented in Fig. 3b, we propose the following equation.

2 $$EF={a}_{0}+{a}_{1}\frac{{d}^{6}}{{d}^{6}+{d}_{0}^{6}}+{a}_{2}{e}^{-d/{D}_{1}}+{a}_{3}{e}^{-d/{D}_{2}}$$

where *a*_0_ is an arbitrary constant that displaces the theoretical values so that the experimental and theoretical values are plotted on the same scale. The second term is the Förster formula that describes the efficiency of excitation energy quenching at short distances; the third term accounts for the near-field decay length with distance away from the AuNR-GaAs interface; and the fourth term accounts for the net carrier diffusion length (rate of diffusion in the bulk GaAs and capture by the QD). The coefficients *a*_*n*_(*n* = 1 − 3) are amplitude adjustment constants. The parameter values *d*_0_ = 3.9 nm (Förster radius), *D*_1_ = 4.8 nm (near-field decay length), and *D*_2_ = 64 nm (carrier diffusion length) produce a very good fit of Eq. 2 to the average enhancement factor as shown by the blue line in Fig. 3b. It is important to note that the near-field felt by the InGaAs (and hence the electron-hole generation) increases exponentially with decreasing GaAs thickness according to the third term in equation (2). The spacer layer thickness that gives maximum enhancement is determined by the competition between the Forster quenching and the near-field enhancement terms. The physical relevance of the parameters in equation (2) is further justified by reproducing the observed emission spectra theoretically in the absence and presence of the AuNRs, and accounting for the emission enhancement due to direct near-field excitation of the InAs/InGaAs and due to carrier diffusion as discussed next. Further experimental data that confirms the contribution of carrier diffusion is presented.

The net emission rate in the illumination area for all the QDs ($\tilde{R}$) results from the competition between the rates of spontaneous emission (*R*) and excitation energy quenching (*Q*), and can be calculated as $\tilde{R}=\sigma (R-Q)$, where *σ* is the areal density of the quantum dots. The net emission spectrum of the quantum dots in the region where the AuNRs are dispersed can be expressed as

3 $$\frac{d\tilde{R}(\omega )}{d\omega }=\sigma \{\eta [\frac{dR(\omega )}{d\omega }-\frac{dQ(\omega )}{d\omega }]+(1-\eta )[\frac{d{R}^{0}(\omega )}{d\omega }-\frac{d{Q}^{0}(\omega )}{d\omega }]\}$$

where *η* is the fraction of the area covered by the AuNRs, and *R* ^0^ and *Q* ^0^ represent the emission and quenching rates, respectively, of the QDs in the absence of the AuNRs. The details of the calculations are provided in the Supplementary Information along with the relevant refs 25, 26. In agreement with the experimental observation, for a certain range of GaAs thickness, the theoretical calculation indicates enhanced emission intensity in the presence of AuNRs as illustrated in Fig. 3c. Plotting the ratio of the peak intensities with respect to the GaAs thickness, the theoretical enhancement factor peaks at *d* = 7.5 nm as shown in Fig. 3d, which is in good agreement with the experimental result presented in Fig. 3b.

**Figure 4:**
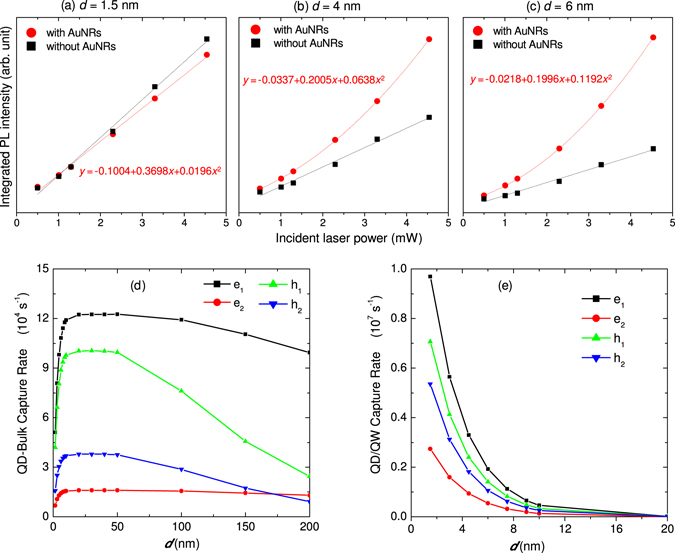
Incident laser power dependence and carrier diffusion. PL plotted as a function of laser power for GaAs thicknesses of (**a**) 1.5 nm, (**b**) 4 nm, and (**c**) 6 nm in the presence (red circles) and absence (black squres) of AuNRs. In the absence of the AuNRs, the PL intensity has a linear dependence on the incident power for all GaAs thicknesses. In the presence of the AuNRs, the intensity varies with laser power quadratically, and the degree of nonlinearity increases with the thickness of GaAs as can be seen by comparing the relative values of the coefficients of the linear and the quadratic terms in (**a**) to (**c**). (**d**) Calculated capture rates by two lowest electron (**e**) and hole (**h**) energy levels of a QD for electrons and holes generated inside GaAs as a function of *d* (GaAs thickness). (**e**) Calculated distance dependence of capture rates by two lowest QD electron and hole levels generated inside the InGaAs quantum well (the trend shows near-field enhanced electron-hole generation inside InGaAs).

As mentioned above, enhanced electron-hole generation at the AuNR-GaAs interface, carrier diffusion through the bulk GaAs, direct excitation of the InGaAs quantum well and carrier capture by the InAs QDs are suggested as key processes that lead to enhanced photon emission by the QDs. Experimental evidence about the emission enhancement mechanism can be obtained by analyzing the PL intensity dependence on the incident laser power for different thicknesses of GaAs as presented in Fig. 4a–c. For a thickness of 1.5 nm, the intensity is lower in the presence of AuNRs because of the dominance of excitation energy transfer from the QD to the metal surface that results in reduced QD PL intensity. However, in both cases (in the absence and presence of AuNRs), the PL intensity increases linearly with the incident laser power as shown in Fig. 4a. When the thickness of the GaAs is increased to 4 nm (Fig. 4b) and 6 nm (Fig. 4c), the PL intensity in the presence of the AuNR increases with laser power quadratically, while in the absence of the AuNRs, the trend remains linear for all thicknesses. Fitting a second order polynomial equation to the data, it can be seen that the quadratic character increases with increasing GaAs thickness as can be quantified from the relative values of the coefficients shown on the corresponding figures. The high degree of nonlinear dependence of the PL intensity on laser power for thicker GaAs capping layer clearly indicates that the observed emission enhancement can be attributed to near-field enhanced exciton generation at the AuNR-GaAs interfaces, and the GaAs capping layer is mediating the exciton-plasmon coupling between the AuNR and InAs QDs through carrier diffusion, capture and recombination processes.

The carrier diffusion and capture processes have been theoretically investigated by describing the temporal evolution of the electron-hole occupation number (*N*) of the QD excited states using a dynamical equation and including the plasmon near-field effect on the absorption and emission property of the QDs^25, 27^. For the first excited state of a QD, the temporal evolution of the occupation number (*N*_1_) is described as

4 $$\frac{d{N}_{1}^{\alpha }}{\partial t}=\frac{{\beta }_{1}({\rm{\Omega }},{\rm{t}}){I}_{0}({\rm{\Omega }},d)}{\hslash {\rm{\Omega }}}-{ {\mathcal R} }_{1}(t)+[1-{N}_{1}^{\alpha }(t)][{\gamma }_{1}^{\alpha }(t)+{\kappa }_{1}^{\alpha }(t)+\sum _{m=2}^{M}\frac{{N}_{m}^{\alpha }(t)}{{\tau }_{0}}]$$

where α represents electrons or holes, Ω is the angular frequency of the incident photons, *I*_0_(Ω, *d*) is the electric field intensity experienced by the QD at thickness *d*, $\hslash =\frac{h}{2\pi }$ (where *h* is Planck’s constant), *β* is the absorption coefficient of the QD, ${\mathcal R}$ is the rate of spontaneous emission, *γ* and *κ* are the rate of capturing carriers from the bulk GaAs and the InGaAs well by the QD, respectively, and *m* = 1, 2, …, *M* are quantum numbers of all the bound energy states. The details of the calculation, including, the dynamical equation for the second and higher excited states are provided in the Supplementary Information. The capture rates of the carriers by the QD from the bulk GaAs and InGaAs well are plotted as a function of GaAs thickness in Fig. 4d and e, respectively. As shown in Fig. 4d, the carrier capture rate from the bulk GaAs decreases rapidly for *d* < 10 nm because for this thickness, the contribution of carrier diffusion is insignificant. The electron-hole (e-h) capture rates remain high up to 50 nm and decreases slowly as the thickness increases further, which is in agreement with the experimentally observed broad carrier diffusion profile in GaAs^28^. In contrast, the direct near-field excitation of InGaAs and the electron and hole capture rates increases exponentially as the GaAs thickness decreases as seen in Fig. 4e. The theoretically calculated result (the trend in Fig. 4e) justifies the physical relevance of the near-field decay length (D_1_) defined in equation (2). Comparing the scales on the y-axes in Fig. 4d and e, it can be seen that the magnitude of the capture rate from the InGaAs layer is significantly higher than that from the bulk GaAs, suggesting that the apparent emission enhancement trend is mainly determined by the carrier capture rate from the immediate capping layer. However, as shown in Fig. 3d, the decay length of the emission enhancement is longer than that of the carrier capture rate from the InGaAs layer (Fig. 4d), indicating that the net enhancement effect results from the contribution of the capture rates from the InGaAs and GaAs layers. Overall, the trend obtained from the theoretical calculations is in accordance with the parameterized equation (2), and further confirms the importance of the carrier capture rates from the InGaAs and GaAs in determining the observed enhanced photon emission by the InAs QDs. We note that the experimental decay length of the emission enhancement (Fig. 3b) is longer than that of the theoretical decay length (Fig. 3d). Our theoretical modeling neglects the capture of carriers by the InGaAs quantum well from the GaAs layer, which becomes significant when direct excitation of the InGaAs quantum well is not dominant. This could enhance the diffusion current in the GaAs layer and slow down the rapid decay of the theoretical *EF* presented in Fig. 3d.

## Conclusion

In summary, we have presented the first systematic study of enhanced localized exciton generation, carrier diffusion and recombination experimentally and theoretically. Using GaAs as an active mediator of the interaction between plasmonic nanostructures and quantum dots embedded in an InGaAs capping layer, we have observed a distance dependent emission enhancement that is attributed to enhanced exciton generation at the metal-semiconductor interfacial regions. The experimental observations are reproduced theoretically describing the temporal evolution of charge carriers in the first and second excited states. The length scales of the near-field enhanced exciton generation, the Förster energy transfer that leads to excitation energy transfer to the metal surface at short distances, the electron and hole capture rates by the quantum dots from the bulk GaAs and InGaAs capping layers are determined. The result presented here has a potential for opening new research directions and may lead to improved detection efficiency using the optical properties of near-field enhancement effect of plasmonic nanoparticle.

## Methods

### Fabrication and integration of materials

The InAs/InGaAs/GaAs semiconductor materials are grown using a molecular beam epitaxy (MBE) reactor on GaAs (001) substrates. First, the GaAs substrate is thermally treated at 630 °C for 20 min to remove the native oxide. The surface is then smoothed by growing a 150 nm thick GaAs at 580 °C. Subsequent growth of InGaAs, InAs and 1.5 nm GaAs at 475 °C results in the InAs QDs confined in the higher band gap InGaAs and GaAs materials. An additional GaAs layer of different thickness is grown (at 580 °C) for studying the distance dependence of the charge carrier generation and diffusion. After removing the excess surfactant from the commercially obtained gold nanorod solution (Nanopartz Inc.) through centrifugation and re-suspension in water, the plasmonic gold nanorods (AuNRs) are dispersed on the GaAs surface by drop-casting and drying the aqueous solution. The AuNRs are applied only on a smaller portion of the sample so that the emission intensity can be compared by recording the photoluminescence (PL) spectra from the two regions (with and without AuNRs).

### Far-field and near-field optical characterizations

The photoluminescence (PL) spectra of the InAs/InGaAs/GaAs quantum dot- in-a-well are acquired at room temperature using a conventional lock-in technique and a modulated HeNe laser (λ = 632.8 nm, average power of 5 mW) for excitation. Emission from the sample was dispersed by a 0.3 m grating monochromator and detected using a near-infrared detector. The dark-field scattering images of the gold nanorods are obtained using a GX51 Olympus microscope. After the dark-field images are obtained, single particle scattering spectra are recorded by centering the individual gold nanorods in the focus of the collection objective and directing the scatted light into a spectrometer (Isoplane Spectrograph of Princeton Instruments) that is equipped with a thermoelectrically cooled (−75 °C) deep depleted CCD camera.

### Topographic and near-field optical characterizations

The topographic and near-field optical images are obtained using an integrated AFM/Near-field system (Neaspec, GmbH). To avoid distortion of the plasmon mode profiles of the samples, the near-field optical images of the gold nanorods are obtained implementing an orthogonal excitation and detection scheme. That is, the sample is excited with an incident laser (λ = 632.8 nm) that is polarized perpendicular to the AFM tip (Arrow-NCPt) and vertically polarized scattered light is selectively detected as described in ref. 20.

### Electromagnetic simulation

The scattering and near-field experimental results are reproduced in electromagnetic simulation. The electromagnetic simulation is carried out using finite-difference time domain (FDTD) method, which is implemented using a commercial software package (Lumerical Solutions, Inc.). A total-field scattered field source scheme is used to introduce light energy into the simulation region, where the grid size is 0.5 nm for all x-, y- and z-axes.

### Theory

Theoretical formalisms that describe the carrier dynamics in the InAs quantum dots and the photon emission enhancement mechanism are presented in the Supplementary Materials.

## Electronic supplementary material

Active Mediation of Plasmon Enhanced Localized Exciton
