# Supplementary material for: Active Mediation of Plasmon Enhanced Localized Exciton Generation, Carrier Diffusion and Enhanced Photon Emission
Source: Sci Rep. 2017 Apr 13;7:864. doi: 10.1038/s41598-017-00964-5 (PMC5429829; doi:10.1038/s41598-017-00964-5)
Supplement: Supplementary file 1 — Active Mediation of Plasmon Enhanced Localized Exciton [file 41598_2017_964_MOESM1_ESM.pdf]

Supplementary Information

Active Mediation of Plasmon Enhanced Localized Exciton Generation, Carrier Diffusion and Enhanced Photon Emissionn

Sharmin Haq[3,4] Sadhvikas Addamane[2,4] Bijesh Kafle[1,4] Danhong Huang[5] Ganesh Balakrishnan[2,3,4] and Terefe G. Habteyes[1,3,4]

[1]*Department of Chemistry & Chemical Biology*

[2]*Electrical & Computer Engineering*

[3]*Optical Science & Engineering Program*

[4]*Center for High Technology Materials,*

University of New Mexico, Albuquerque, NM 87131, United States

[5]*Space Vehicles Directorate, Air Force Research Laboratory,*

Kirtland AFB, NM 87117, United States

The time evolution for the level occupations N_n^α of a quantum dot is determined for $n = 1$ from

$$\frac{dN_1^\alpha(t)}{dt} = \frac{\beta_1^{\rm QD}(\Omega_{\rm sp},\,t)\,I_0(\Omega_{\rm sp},\,d)}{\hbar\Omega_{\rm sp}} - \mathcal{R}_1^{\rm QD}(t)$$

$$+\left[\gamma_1^\alpha(t)+\kappa_1^\alpha(t)\right]\left[1-N_1^\alpha(t)\right] + \sum_{m=2}^{M}\frac{N_m^\alpha(t)}{\tau_0}\left[1-N_1^\alpha(t)\right]\ , \tag{1}$$

and for $n \geq 2$ from

$$\frac{dN_n^\alpha(t)}{dt} = \frac{\beta_n^{\rm QD}(\Omega_{\rm sp},\,t)\,I_0(\Omega_{\rm sp},\,d)}{\hbar\Omega_{\rm sp}} - \mathcal{R}_n^{\rm QD}(t)$$

$$+\left[\gamma_n^\alpha(t)+\kappa_n^\alpha(t)\right]\left[1-N_n^\alpha(t)\right] + \sum_{m=n+1}^{M}\frac{N_m^\alpha(t)}{\tau_0}\left[1-N_n^\alpha(t)\right] - \sum_{m=1}^{n-1}\frac{N_n^\alpha(t)}{\tau_0}\left[1-N_m^\alpha(t)\right]\ , \tag{2}$$

where α = e or h corresponds to electrons or holes in a quantum dot, $n = 1,\,2,\,\cdots\,,\,M$ label all the bound-state energy levels, $\hbar\Omega_{\rm sp}$ is the energy of illuminating photons, d is the spacer-layer thickness, τ_0 is the inter-level energy-relaxation time, $\mathcal{R}_n^{\rm QD}(t)$ and $\beta_n^{\rm QD}(\Omega_{\rm sp},\,t)$ are the nth-level associated rate for spontaneous emission and absorption coefficient, $\gamma_n^\alpha(t)$ and $\kappa_n^\alpha(t)$ are the capture rates for photo-excited carriers from the spacer-layer and from the quantum well. The detailed calculations of $\mathcal{R}_n^{\rm QD}(t)$, $\beta_n^{\rm QD}(\Omega_{\rm sp},\,t)$, $\gamma_n^\alpha(t)$, $\kappa_n^\alpha(t)$, and $I_0(\Omega_{\rm sp},\,z)$ are presented in Appendix A.

Including the reabsorption of the quantum-dot spontaneous emission by metal nanorods on the surface of the spacer layer, we get the net rate per area from all quantum dots $\tilde{R}_{\rm QD} = \sigma_{\rm QD}(R_{\rm QD} - Q_{\rm QD})$, where $\sigma_{\rm QD}$ is the quantum-dot areal density. The quenched quantum-dot emission spectrum is given by

$$\frac{d\tilde{R}_{\rm QD}(\omega)}{d\omega} = \sigma_{\rm QD}\left\{\eta_{\rm NR}\left[\frac{dR_{\rm QD}(\omega)}{d\omega} - \frac{dQ_{\rm QD}(\omega)}{d\omega}\right]\right.$$

$$\left.+(1-\eta_{\rm NR})\left[\frac{dR_{\rm QD}^{(0)}(\omega)}{d\omega} - \frac{dQ_{\rm QD}^{(0)}(\omega)}{d\omega}\right]\right\}\ , \tag{3}$$

where $\eta_{\rm NR}$ is the surface-filling fraction of metallic nanorods, the first and second terms correspond to the emission and the quenching spectra with illumination in the region with and without nanorods, respectively. Additionally, we calculate the emission and quenching spectra as [1, 2]

$$\frac{dR_{\rm QD}(\omega)}{d\omega}=\left(\frac{\sqrt{\epsilon_{\rm b}}\,e^2\hbar^2}{4\pi^2\mu_{\rm r}'\epsilon_0\epsilon_{\rm b}}\right)\left[\varepsilon_1^{\rm e}+\varepsilon_1^{\rm h}+\varepsilon_{\rm G}(T)\right]\frac{\omega^3}{c^3}$$

$$\times\sum_{n=1}^{M}N_n^{\rm e}\,N_n^{\rm h}\left\{\frac{\Theta[\hbar\omega-\varepsilon_n^{\rm e}-\varepsilon_n^{\rm h}-\varepsilon_{\rm G}(T)]}{[\varepsilon_n^{\rm e}+\varepsilon_n^{\rm h}+\varepsilon_{\rm G}(T)]^2}\right\}\frac{\hbar\delta_n}{[\hbar\omega-\varepsilon_{\rm G}(T)-\varepsilon_n^{\rm e}-\varepsilon_n^{\rm h}]^2+\hbar^2\delta_n^2}\ , \tag{4}$$

$$\frac{dQ_{\rm QD}(\omega)}{d\omega}=-\eta_{\rm NR}\left(\frac{9\kappa_{\rm QD}^2\Delta Z\mathcal{S}_{\rm NR}}{8\pi\epsilon_{\rm b}^2\,d^6}\right){\rm Im}\left[\epsilon_{\rm NR}(\omega)\right]\frac{c^3}{\omega^3}\left[\frac{dR_{\rm QD}(\omega)}{d\omega}\right]\ , \tag{5}$$

where $\Theta(x)$ is the unit-step function, $\hbar\omega$ is the energy of emitted photons, $\mu_{\rm r}'$ is the reduced e-h mass in a quantum dot, $\varepsilon_{\rm G}(T)$ and $\epsilon_{\rm b}$ are the bandgap energy and dielectric constant of the host material for quantum dots, $\varepsilon_n^{\rm e}$ and $\varepsilon_n^{\rm h}$ are the nth energy levels of electrons and holes, $\hbar\delta_n$ is the homogeneous level broadening of the quantum dot, ΔZ and $\mathcal{S}_{\rm NR}$ are the thickness and surface area of a nanorod, $\kappa_{\rm QD}^2$ is the orientation function between dipoles of a quantum dot and a nanorod, and ${\rm Im}\,[\epsilon_{\rm NR}(\omega)]$ is the nanorod loss function [3].

Appendix A: Photo-Carrier Dynamics in Quantum Dot

The quantities, $\mathcal{R}_n^{\rm QD}$, $\beta_n^{\rm QD}$, γ_n^α, κ_n^α and $I_0(\Omega_{\rm sp},\,z)$, introduced in Eqs. (1) and (2) are calculated as [1, 4, 5]

$$\mathcal{R}_n^{\rm QD}(t)=\left(\frac{\sqrt{\epsilon_{\rm b}}\,e^2}{4\pi\mu_{\rm r}^\prime c^3\epsilon_0\epsilon_{\rm b}\hbar^2}\right)\left[\varepsilon_{\rm G}(T)+\varepsilon_1^{\rm e}+\varepsilon_1^{\rm h}\right]\left[\varepsilon_{\rm G}(T)+\varepsilon_n^{\rm e}+\varepsilon_n^{\rm h}\right]N_n^{\rm e}(t)N_n^{\rm h}(t)\ , \tag{A1}$$

$$\beta_n^{\rm QD}(t)=\frac{\Omega_{\rm sp}\sqrt{\epsilon_{\rm b}}}{c}\left(\frac{2e^2}{\epsilon_0\epsilon_{\rm b}}\right)\frac{[\varepsilon_{\rm G}(T)+\varepsilon_1^{\rm e}+\varepsilon_1^{\rm h}]\hbar^2}{4\mu_{\rm r}^\prime}\left(\frac{|\langle\psi_n^{\rm e}|\psi_n^{\rm h}\rangle|^2}{[\varepsilon_{\rm G}(T)+\varepsilon_n^{\rm e}+\varepsilon_n^{\rm h}]^2}\right)$$
$$\times\frac{[1-N_n^{\rm e}(t)-N_n^{\rm h}(t)]\,\hbar\delta_n}{[\hbar\Omega_{\rm sp}-\varepsilon_{\rm G}(T)-\varepsilon_n^{\rm e}-\varepsilon_n^{\rm h}]^2+\hbar^2\delta_n^2}\ , \tag{A2}$$

$$\gamma_n^\alpha(t)=\mathcal{S}_n\left[\frac{n_\alpha(d,\,t)\,e^2\ell_0^2}{\epsilon_0}\right]\frac{\beta_{\rm HR}}{\hbar}\left(\frac{1}{\epsilon_\infty}-\frac{1}{\epsilon_{\rm s}}\right)e^{-[2N_{\rm ph}(\Omega_0)+1]\beta_{\rm HR}}$$
$$\times\left[1-\frac{\Delta E_n^\alpha}{\hbar\Omega_0\beta_{\rm HR}}\right]^2\exp\left[\frac{\Delta E_n^\alpha}{2k_BT}\right]\sum_{m=1}^\infty\frac{(\delta_n/\Omega_0)\,I_m(\xi)}{[m-\Delta E_n^\alpha/\hbar\Omega_0]^2+(\delta_n/\Omega_0)^2}\ , \tag{A3}$$

$$\kappa_n^\alpha(t)=\mathcal{S}_n\left[\frac{\sqrt{\pi}[2^{n-1}(n-1)!]n_{\rm 2D}^\alpha(t)\,e^2\ell_0^2}{2\epsilon_0}\right]\frac{\beta_{\rm HR}}{\hbar}\left(\frac{1}{\epsilon_\infty}-\frac{1}{\epsilon_{\rm s}}\right)e^{-[2N_{\rm ph}(\Omega_0)+1]\beta_{\rm HR}}$$
$$\times\left[1-\frac{\Delta\varepsilon_n^\alpha}{\hbar\Omega_0\beta_{\rm HR}}\right]^2\exp\left[\frac{\Delta\varepsilon_n^\alpha}{2k_BT}\right]\sum_{m=1}^\infty\frac{(\delta_n/\Omega_0)\,I_m(\xi)}{[m-\Delta\varepsilon_n^\alpha/\hbar\Omega_0]^2+(\delta_n/\Omega_0)^2}\ , \tag{A4}$$

$$I_0(\Omega_{\rm sp},\,z)=\eta_{\rm NR}\,\mathcal{I}_0\left(\frac{c^2}{\Omega_{\rm sp}^2}\right)\left[|\beta_0(k_0,\,\Omega_{\rm sp})|^2+|k_0(\Omega_{\rm sp})|^2\right]\,\exp\left\{-2{\rm Re}[\beta_0(k_0,\,\Omega_{\rm sp})]z\right\}\ , \tag{A5}$$

where ψ_n^α is the wavefunction of a quantum dot for electrons or holes, $\mathcal{S}_n$ is a numerical factor, ℓ_0 represents the effective radius of a spherical-shape dot, $\beta_{\rm HR}$ is the Huang-Ryhs factor, ϵ_∞ and $\epsilon_{\rm s}$ are the high-frequency and static dielectric constants of the host semiconductor, $\Delta E_n^{\rm e}=\Delta E_{\rm c}(T)-\varepsilon_n^{\rm e}$, $\Delta E_n^{\rm h}=\Delta E_{\rm v}(T)-\varepsilon_n^{\rm h}$, $\Delta E_{\rm c}(T)$ and $\Delta E_{\rm v}(T)$ are the conduction and valence band offsets of a dot related to the spacer layer, $\Delta\varepsilon_n^{\rm e}=\Delta\varepsilon_{\rm c}(T)-\varepsilon_n^{\rm e}$, $\Delta\varepsilon_n^{\rm h}=\Delta\varepsilon_{\rm v}(T)-\varepsilon_n^{\rm h}$, $\Delta\varepsilon_{\rm c}(T)$ and $\Delta\varepsilon_{\rm v}(T)$ are the conduction and valence band offsets of a quantum dot related to the quantum well, $\hbar\Omega_0$ is the optical-phonon energy, $N_{\rm ph}(\Omega_0)=[\exp(\hbar\Omega_0/k_BT)-1]^{-1}$ is the distribution function of thermal-equilibrium phonons, T is the system temperature, $I_m(\xi)$ is the modified Bessel function of the mth-order with $\xi=2\beta_{\rm HR}\sqrt{N_{\rm ph}(\Omega_0)[N_{\rm ph}(\Omega_0)+1]}$, $n_\alpha(z)$ ($n_{\rm 2D}^\alpha$) is the concentrations (areal density) of photo-excited electrons or holes in the

spacer layer (quantum well), $\mathcal{I}_0 = (\epsilon_0 E_0^2/2)\, c$, E_0 is the amplitude of free-space incident light, and $\beta_0(k_0,\, \Omega_{\rm sp})$ $[k_0(\Omega_{\rm sp})]$ is the transverse (longitudinal) wave vector of an induced surface-plasmon-polariton wave.

The dynamical equations for spacer-layer carrier concentration $n_\alpha(z,\, t)$ and the quantum-well subband density $n_{\rm 2D}^\alpha$ are presented in Appendices B and C below.

Appendix B: Photo-Carrier Dynamics in Spacer Layer

The drift-diffusion equations for photo-excited electrons and holes in a spacer layer is written as [1]

$$\frac{\partial n_\alpha(z,\, t)}{\partial t} = D_\alpha\, \frac{\partial^2 n_\alpha(z,\, t)}{\partial z^2} \mp \mu_\alpha\, \frac{\partial}{\partial z} \left[n_\alpha(z,\, t)\, \mathcal{E}_0(z,\, t)\right] + \frac{\beta_{\rm abs}^{\rm 3D}(\Omega_{\rm sp},\, z) I_0(\Omega_{\rm sp},\, z)}{\hbar\Omega_{\rm sp}} - \mathcal{R}_{\rm sl}^{\rm 3D}(z,\, t) - \left.\frac{n_\alpha(z,\, t)}{T'_\alpha}\right|_{z=d} . \tag{B1}$$

where the signs $-$ and $+$ correspond to holes and electron, respectively, $D_\alpha = (k_B T/e)\, \mu_\alpha$ is the diffusion coefficient of photo-carriers, $\mu_\alpha = e\tau_\alpha/m_\alpha^*$ is the carrier mobility, τ_α is the momentum-relaxation time, m_α^* are the effective masses of spacer-layer electrons and holes, $\beta_{\rm abs}^{\rm 3D}(\Omega_{\rm sp},\, z)$ is the absorption coefficient, $\mathcal{R}_{\rm sl}^{\rm 3D}(z,\, t)$ is the spontaneous emission of photons, and $1/T'_\alpha$ represents the decay rate by carrier scattering between the spacer layer and quantum well at $z = d$. The space-charge field $\mathcal{E}_0(z,\, t)$ can be determined from the Poisson's equation:

$$\frac{\partial \mathcal{E}_0(z,\, t)}{\partial z} = \frac{e}{\epsilon_0 \epsilon_{\rm b}} \left[n_{\rm h}(z,\, t) - n_{\rm e}(z,\, t)\right] . \tag{B2}$$

Moreover, the boundary conditions are given by

$$D_\alpha\, \left.\frac{\partial n_\alpha(z,\, t)}{\partial z}\right|_{z=0} = 0 \;, \qquad \left.\mathcal{E}_0(z,\, t)\right|_{z\leq 0} = 0 \;, \tag{B3}$$

$$-\, D_\alpha\, \left.\frac{\partial n_\alpha(z,\, t)}{\partial z}\right|_{z=d} + \theta[\pm\mathcal{E}_0(z,\, t)] |\mathcal{E}_0(z,\, t)| \mu_\alpha n_\alpha(z,\, t)|_{z=d} = \sigma_{\rm QD} \sum_{n=1}^{M} \gamma_n^\alpha \left[1 - N_n^\alpha(t)\right] \;, \tag{B4}$$

where the signs $+$ and $-$ correspond to holes and electrons, respectively.

The calculations of both $\beta_{\rm abs}^{\rm 3D}(\Omega_{\rm sp},\, z)$ and $\mathcal{R}_{\rm sl}^{\rm 3D}(z)$ give rise to [1, 6, 7]

$$\beta^{3D}_{abs}(\Omega_{sp},\,z)=\frac{\Omega_{sp}\sqrt{\epsilon_b}}{c}\left\{\frac{\gamma_0\Omega^2_{pl}(z)}{\Omega_{sp}(\Omega^2_{sp}+\gamma_0^2)}\right.$$

$$\left.+\frac{e^2\hbar^2}{8\pi\mu_r\epsilon_0\epsilon_b E_G(T)}\left(\frac{2\mu_r}{\hbar^2}\right)^{3/2}\theta[\hbar\Omega_{sp}-E_G(T)]\,[\hbar\Omega_{sp}-E_G(T)]^{1/2}\right\}\ , \tag{B5}$$

$$\mathcal{R}^{3D}_{sl}(z,\,t)=n_e(z,\,t)n_h(z,\,t)\,\frac{e^2\hbar\,(2\pi\mu_r\epsilon_b k_B T)^{1/2}}{32(2\pi)^6(m^*_e m^*_h c^2)^{3/2}\epsilon_0\epsilon_b}$$

$$\times\left\{105+\frac{90E_G(T)}{k_BT}+\left[\frac{6E_G(T)}{k_BT}\right]^2+\left[\frac{2E_G(T)}{k_BT}\right]^3\right\}\ , \tag{B6}$$

where $\hbar\gamma_0$ is the homogeneous level broadening, $\mu_r=(1/m^*_e+1/m^*_h)^{-1}$ is the reduced e-h mass, $\Omega^2_{pl}(z)=(e^2/\epsilon_0\epsilon_b)\,[n_e(z)/m^*_e+n_h(z)/m^*_h]$ is the frequency of the e-h composite plasma, [1] and $E_G(T)$ is the bandgap of the spacer layer.

Moreover, the decay rate by carrier scattering between the spacer layer and quantum well at $z=d$ is calculated as

$$\frac{1}{T'_{e,h}}=\frac{\Omega_0 e^2}{256\pi\epsilon_0}\,[N_{ph}(\Omega_0)+1]\left(\frac{1}{\epsilon_\infty}-\frac{1}{\epsilon_s}\right)\sqrt{\frac{2M^*_{e,h}}{\pi\hbar^2[\Delta\varepsilon''_{c,v}(T)-E^{e,h}_1-\hbar\Omega_0]}}$$

$$\times\left\{4-3\left[\frac{k_BT}{\Delta\varepsilon''_{c,v}(T)-E^{e,h}_1-\hbar\Omega_0}\right]\right\}\ . \tag{B7}$$

Appendix C: Photo-Carrier Dynamics in Quantum Well

The subband density of photo-excited electrons and holes in a quantum well is derived as

$$\frac{dn^\alpha_{2D}(t)}{dt}=\frac{\beta^{qw}_{abs}(\Omega_{sp})L_W I_0(\Omega_{sp},\,d)}{\hbar\Omega_{sp}}-\mathcal{R}_{qw}(t)-\sigma_{QD}\sum_{n=1}^{M}\kappa^\alpha_n(t)\,[1-N^\alpha_n(t)]+\frac{n_\alpha(z,\,t)L_W}{T'_\alpha}\ , \tag{C1}$$

where L_W is the quantum-well width,

$$\beta^{qw}_{abs}(\Omega_{sp})=\frac{\Omega_{sp}\sqrt{\epsilon_b}}{c}$$

$$\times\left\{\frac{\gamma_0\Omega^2_{pl}}{\Omega_{sp}(\Omega^2_{sp}+\gamma_0^2)}+\frac{e^2}{4\epsilon_0\epsilon_b L_W E'_G(T)}\,\theta\left(\hbar\Omega_{sp}-E'_G(T)-E^e_1-E^h_1\right)\right\}\ , \tag{C2}$$

$$\mathcal{R}_{\rm qw}(t) = \frac{e^2}{c^3\epsilon_0\sqrt{\epsilon_{\rm b}}}\,n_{\rm 2D}^{\rm e}(t)n_{\rm 2D}^{\rm h}(t)\left[\frac{(k_{\rm B}T)^2}{4M_{\rm e}^*M_{\rm h}^*E'_{\rm G}(T)}\right]$$
$$\times\left[6+6\left(\frac{E'_{\rm G}(T)}{k_{\rm B}T}\right)+3\left(\frac{E'_{\rm G}(T)}{k_{\rm B}T}\right)^2+\left(\frac{E'_{\rm G}(T)}{k_{\rm B}T}\right)^3\right]\ , \tag{C3}$$

$E'_{\rm G}(T) = \bar{E}_{\rm G}(T) + E_1^{\rm e} + E_1^{\rm h}$, $\bar{E}_{\rm G}(T)$ is the bandgap of the quantum-well host material, $M_{\rm e}^*$ ($M_{\rm e}^*$) is the electron (hole) effective mass, and $E_1^{\rm e}$ ($E_1^{\rm h}$) is the subband bandedge of electrons (holes) in the quantum well.

---

[1] D. H. Huang and P. M Alsing, Phys. Rev. B **78**, 035206 (2008).

[2] D. L. Andrews, "Resonance energy transfer: theoretical foundations and developing applications" in *Tutorials in Complex Photonic Media* (SPIE Press, Bellingham, WA, pp. 439-478, 2009).

[3] X. Fan, W. Zheng, and D. J. Singh, Light: Science & Applications **3**, e179 (2014).

[4] D. H. Huang, M. M. Easter, G. Gumbs, A. A. Maradudin, S.-Y. Lin, D. A. Cardimona, and X. Zhang, Opt. Expr. **22**, 27576 (2015).

[5] D. H. Huang, F. Gao, D. A. Cardimona, C. P. Morath, and V. M. Cowan, Am. J. Space Sci. **3**, 3 (2015).

[6] S. K. Lyo, Phys. Rev. B **73**, 205322 (2006).

[7] S. K. Lyo and E. D. Jones, Phys. Rev. B **38**, 4113 (1988).
